# 

**Published:** 1885-07-20

**Authors:** 


### TABLE OF CONTENTS.

ORIGINAL AND SELECTED ARTICLES:

Anteflexion of the Uterus and its Associated Pathological Conditions—their Prevention and Treatment, by G. G. Roy, M.D., Prof. Materia Medica, etc.. So. Med. Col, 241. Enteralgia—A Rare Case, by Johan S. Carothers, M.D, of Mississippi, 248 Bromide of Ethyl as an Anæsthetic in Labor. 250. The Negro Exempt from Hay Fever, 253. A Case of Opium Poisoning Successfully Treated with Atropine and Caffeine by Chas. N. Dixon Jones, B S., M.D.. Brooklyn, Late House Surgeon to the Brooklyn Hospital, 254. Chicago Gynæcological Society, 256. Liquor Ipecacuanhæ et Morphinæ—Dover's Solution, by Edwin H Hess, Ph. G., 257. Buttermilk in Sick Stomach, by R. J. Pere, M. D., Pleasanton, Kan., 259 A Note on the Management of Shoulder Presentations, by E F. Wells M.D., Minster, Ohio, 260 Two Cases of Traumatic Tetanus, by H. Spier, M.D., Jordan, Minn., 261. Rapid Anæthesia, by Ether; Goldbaum, 262.

ABSTRACTS AND GLEANINGS:

Hemorrhagic Malarial Fever, 263. The Therapeutical Value of Arsenic in Anæmia and Atrophic Conditions, 264. Summer Complaints of Children, 265. The Treatment of Carbuncle Without Incision, 266 Fighting Cholera by Inoculation, 267. Hints on the Usage of Drainage Tubes, 268. Goitre Successfully Treated; Medical Prizes; Health of the Chinese, 269. New Method of Treating Gonorrhœa; Water for Infants; Vaccination with Fever Virus, 270.

SCIENTIFIC ITEMS:

An Educated Chimpanzee; Industrial Use of Natural Gas, 271. Rates of Increase of Negro Population; The Medical Electric Lamp, 272.

PRACTICAL NOTES AND FORMULÆ.

Coffee Extract; Clymer's Mixture for Asthma; A Mixture for Whooping Cough; Lyon's Kathairon, 273. Sedative Cough Mixture; Lotions for Pruritus of the Genitals; Cocaine in the Treatment of Sore Nipples; Compound Carbolic Ointment; Glycerinum Aluminis, 274.

EDITORIALS AND MISCELLANEOUS.

Editorial Notices; Southern Medical College; Typhoid Epidemic; " Don't view me with a Rabbit's Eye;" Peacock's Fucus Marina, and Peacock's Bromides; The Mississippi Valley Medical Society, 275. That Cancer Case; In Memoriam, 276. Books and Pamphlets received, 277. Receiped; Special Notices, 280.

